# Early mobilization after lung transplantation: A scoping review protocol

**DOI:** 10.1016/j.mex.2025.103404

**Published:** 2025-05-29

**Authors:** Matthieu Reffienna, Adéla Foudhaïli, Colin Sidre, Damien Vitiello, Jonathan Messika

**Affiliations:** a Service de Réanimation polyvalente, Hôpital Foch, Suresnes, France; b Université Paris Cité, Inserm, U1149 CRI, Paris, France; c Université Paris Cité, URP3625 I3SP, Paris, France; d Université Paris Cité, Inserm, U942 MASCOT, Paris, France; e Département de Médecine Physique et Réadaptation, CHU Lariboisière, AP-HP, Paris, France; f Université Paris Cité, DGDBM, BIU Santé médecine, Paris, France; g Université Paris Cité, UFR STAPS, Paris, France; h Unité de Soins Intensifs Respiratoires, Hôpital Foch, Suresnes, France; i Paris Transplant Group, Paris, France

**Keywords:** Critical care, Lung transplantation, Physiotherapy, Rehabilitation, Scoping review protocol.

## Abstract

After lung transplantation, rehabilitation is part of patients’ standard of care, playing a crucial role in enhancing functional capacity and quality of life. Although early rehabilitation in the intensive care unit has been reported in few studies, it is not yet widely standardized in clinical practice. As a result, rehabilitation protocols are often initiated several weeks after surgery, potentially leaving a gap in early postoperative management. While the benefits of early mobilization in critically ill patients are well documented, limited data exist on mobilization strategies initiated immediately in the intensive care unit following lung transplantation. Understanding the feasibility, benefits, and potential barriers to early mobilization in this population would enhance future post-transplant recovery care.

Studies evaluating an early mobilization (<7d) protocol initiated in the intensive care unit for adult lung transplant recipients will be included. Studies assessing protocols initiated later (>7d) in the recovery process will be excluded. A comprehensive search will be conducted in MEDLINE (PubMed), Embase, PEDro, Web of Science Core Collection, CINAHL (EBSCOhost), Scopus, Cochrane CENTRAL, and PROSPERO, as well as relevant gray literature sources. No language or date restrictions will be applied. Study selection and data extraction will be performed independently by two reviewers.

• The main question is: what evidence exists regarding the feasibility, safety, and clinical outcomes of early mobilization interventions for LTx recipients in critical care settings?

## Graphical abstract

**Figure:**
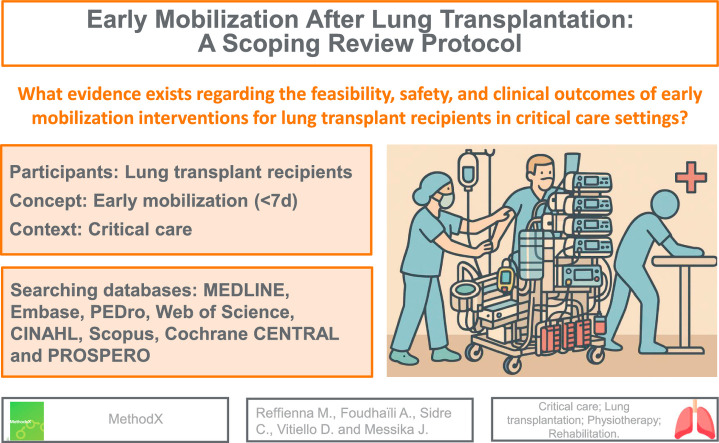

Specifications table

**Table:** 

Subject area	Medicine and Dentistry
More specific subject area	Physiotherapy
Name of your protocol	Early Mobilization After Lung Transplantation: A Scoping Review Protocol
Reagents/tools	Not applicable.
Experimental design	Not applicable.
Trial registration	Open Science Framework https://osf.io/nhr3e
Ethics	This scoping review will utilize data from previously published studies and does not involve human participants, animal experiments, or data collected from social media platforms. The authors commit to maintaining strict vigilance regarding the origin of organs used in research and clinical practice to ensure compliance with human rights and ethical standards
Value of the Protocol	• A comprehensive literature review may offer valuable insights into existing clinical practices and support the development of strategies aimed at optimizing the initial management of lung transplant recipients in critical care settings. • A preliminary search of PROSPERO revealed no ongoing systematic or scoping reviews on this specific topic. In addition, a search of MEDLINE and the Cochrane Database of Systematic Reviews identified no published reviews addressing this question. • Considering the anticipated limited number of studies and the likely low level of evidence, a scoping review was deemed more suitable than a meta-analysis.

## Background

Lung transplantation (LTx) is performed on >4500 patients worldwide each year in order to improve survival and quality of life [1] of patients with end-stage respiratory failure [2]. Underlying conditions leading to LTx include chronic obstructive pulmonary disease, bronchiectasis and idiopathic interstitial pneumonia [2] while cystic fibrosis represent nowadays a marginal indication [3].

Patients with advanced-stage respiratory disease are often sedentary and exhibit severe exercise limitations [4,5]. Moreover, the increasing age of LTx candidates and the frequent use of corticosteroids contribute to sarcopenia. Consequently, there is substantial evidence supporting the necessity and benefits of prehabilitation programs while awaiting LTx [6]. These protocols are numerous, well-defined, and their outcomes encourage their implementation in LTx candidates [7].

Postoperatively, rehabilitation protocols remain essential components of patient care. Robust data demonstrated that such interventions could improve muscle strength, functional performance, and quality of life [6]. However, rehabilitation programs are typically initiated 4 to 6 weeks after lung transplantation, once patients are medically stable [8]. While the optimal period for natural recovery is within the first three months following LTx [9,10], it is crucial not to delay the initiation of these protocols. Rehabilitation should begin as soon as feasible, starting with early mobilization in the intensive care unit

The LTx postoperative period is a critical phase, with numerous conditions that may hinder early mobilization. The main identified limitations and complications in the intensive care unit (ICU) include [9,11,12]

- Medical condition: hemodynamic stability, invasive or non-invasive mechanical ventilation, respiratory failure, neurological impairment, frequent use of ECMO to manage severe primary graft dysfunction;
- Daily medical and nursing care, such as bronchoscopic procedures, wound care, plasma exchanges;
- Invasive devices, such as chest drains, central venous and arterial catheters, epidural analgesia catheters;
- Non-infectious complications: pain, acute rejection, pleural effusion, bronchial dehiscence, gastroparesis, arrhythmias, swallowing disorders;
- Infectious complications: such as nosocomial or opportunistic infections.

Patients in ICU are also at risk of developing intensive care unit-acquired weakness (ICU-AW), a syndrome involving motor nerve fiber damage and muscle catabolism, which can result in flaccid quadriplegia [13]. In all-cause ICU patients, muscle catabolism can reach up to 2 % per day of hospitalization [14]. ICU-AW contributes to increased morbidity and mortality both during and after hospitalization [15,16]. Risk factors include sepsis and immobility [13].

Early mobilization has been investigated in >4500 studies over the past 15 years. It is recommended by numerous scientific societies as part of ICU patients’ care bundles [17,18]. However, considerable variability in protocols and their short- and long-term effects have been reported. Nonetheless, when properly conducted, early mobilization has been shown to be safe for patients in ICU, adverse events occurring in <3 % of cases [19].

Therefore, implementing early mobilization protocols in ICU following LTx appears relevant, both to mitigate ICU-AW and its consequences and to improve patients’ functional prognosis. However, due to the complexity of post-LTx care and the relatively limited number of patients, data on this topic are scarce [[20], [21], [22], [23], [24], [25]].

A literature review could provide an overview of current practices, enabling the proposal of strategies to optimize the initial care of LTx recipients in the critical care settings. Given the expected low number of articles with limited evidence levels, a scoping review was considered more appropriate than a meta-analysis.

### Description of protocol

The main question is: what evidence exists regarding the feasibility, safety, and clinical outcomes of early mobilization interventions for lung transplant recipients in critical care settings?

Sub-questions are:

- What is the reported time-frame between the LTx and the beginning of rehabilitation interventions?
- What have been the early mobilization techniques already assessed?
- Which outcomes have been targeted by these interventions?
- What adverse effects/events related to these interventions have been studied or reported?
- Which barriers to early mobilization have been identified?
- Which gaps in the scientific literature need to be filled on the topic of early mobilization interventions following LTx?

### Participants

This scoping review will include all studies focusing on adult LTx recipients (> 18 years). Patients who have undergone combined transplants (e.g., heart-lung transplantation) will be excluded. No exclusions will be made based on the underlying condition leading to transplantation, the surgical techniques (single or double-side LTx) or whether patients were on high emergency conditions. Animal models of transplantation will also be excluded.

### Concept

The concept of the scoping review is: early mobilization. All articles evaluating a mobilization protocol initiated in the immediate postoperative period of LTx (< 7days) will be included. No restrictions will be applied regarding the type of mobilization used (e.g., bed mobilization, sitting in a chair, verticalization, walking, electrostimulation). Studies involving patients supported with ECMO will not be excluded. Studies focusing strictly on respiratory protocols (e.g., airway clearance or exclusive respiratory muscle strengthening) will be excluded. However, if the protocols are mixed, the studies will be included, and only the motor component will be analyzed whenever possible.

### Context

The scoping review will include all studies in which the intervention begins in an ICU and/or intermediate care units. Studies will be excluded if rehabilitation is initiated after the patient’s discharge from critical care or if the intervention begins >7 days after surgery. No geographical restrictions will be applied.

### Types of sources

This scoping review will include a broad range of study designs. Eligible experimental and quasi-experimental studies will encompass randomized controlled trials, non-randomized controlled trials, before-and-after studies, and interrupted time series. Analytical observational studies—such as prospective and retrospective cohort studies, case-control studies, and analytical cross-sectional studies—will also be considered. Additionally, descriptive observational studies, including case series, individual case reports, and descriptive cross-sectional studies, will be included. Conference abstracts, ongoing research protocols, systematic reviews, and clinical guidelines that address relevant interventions and meet the inclusion criteria will be incorporated as well. Texts and opinion papers will also be considered within the scope of this review.

### Search strategy

The search strategy is designed to capture both published and unpublished literature. An initial limited search of EMBASE and MEDLINE was conducted to identify studies related to early mobilization following LTx. Keywords from the titles and abstracts of relevant articles, along with the associated index terms, were used to construct a comprehensive search strategy for MEDLINE via PubMed (see Table 1). This strategy, including all identified keywords and indexing terms, will be adapted for each selected database and information source. To ensure thoroughness, both forward and backward citation tracking (snowballing and reverse snowballing) will be performed to identify additional relevant studies. The initial limited search, final search strategy, and database selection were developed in collaboration with a medical librarian (CS).

**Table 1:** Search strategy for MEDLINE (via pubmed), conducted on February 18, 2025.

Search	Query	Records retrieved
#1	"lung transplantation"[MeSH Terms] OR "lung transplant*"[TIAB] OR "pulmonary transplant*"[TIAB] OR "lung graft*"[TIAB] OR "pulmonary graft*"[TIAB] OR "lung allotransplant*"[TIAB]	27,378
#2	"early mobili*"[TIAB] OR "Accelerated Ambulat*"[TIAB] OR "Accelerated mobili*"[TIAB]	5710
#3	"critical care"[MeSH Terms] OR "intensive care units"[MeSH Terms] OR "intensive care"[TIAB] OR "critical care"[TIAB] OR "intensive therap*"[TIAB] OR "ICU"[TIAB] OR "ICUs"[TIAB] OR "recovery room*"[TIAB] OR "acute*"[TIAB] OR "inpatient*"[TIAB] OR "Inpatients"[Mesh] OR "early"[TIAB] OR "immediate"[TIAB]	3990,695
#4	"physiotherap*"[TIAB] OR "Physical Therapy Specialty"[Mesh] OR "physical therapy modalities"[MeSH Terms] OR "physical therap*"[TIAB] OR "rehabilitation"[MeSH Terms] OR "rehabilitation" [Subheading] OR "rehabilitat*"[TIAB] OR "exercice"[TIAB] OR "mobilization"[TIAB]	753,835
#5	#3 AND #4	127,769
#6	#5 OR #2	128.556
#4	#1 AND #6	332

The databases and trial registers to be searched include MEDLINE (PubMed), Embase, CINAHL (EBSCOhost), Web of Science, PEDro, Scopus, Cochrane CENTRAL and PROSPERO.

Studies published in any language will be considered for inclusion. While the review team primarily works in French and English, articles published in other languages will be translated using either the Pro version of DeepL Translator (DeepL, Cologne, Germany) or a validated language model such as ChatGPT (OpenAI, San Francisco, CA, USA), to ensure accurate and context-aware translation. The chosen translation method will be documented in the final manuscript. However, languages using non-Latin alphabets such as Chinese or Cyrillic scripts will be excluded. A detailed list of excluded languages will be provided in the appendix.

### Source of evidence selection

A three-stage screening process will be employed to identify relevant studies. All retrieved citations will first be imported into Rayyan (Qatar Computing Research Institute, Doha, Qatar), where duplicates will be removed. The deduplication threshold will be set at 95 % similarity to identify and eliminate duplicate records.

Following a pilot test of the eligibility criteria, two independent reviewers (MR and AF) will screen the titles and abstracts to assess their relevance based on the predefined inclusion criteria. Full texts of potentially eligible studies will then be retrieved and reviewed in detail by the same two reviewers.

Reasons for the exclusion of studies at the full-text screening stage will be documented and reported in the final scoping review. Any disagreements during the screening process will be resolved through discussion, or, if necessary, with the involvement of additional reviewers (JM and DV). The overall selection process, including search results and study inclusion, will be reported using a PRISMA flow diagram [26].

### Data extraction

Data will be extracted from the studies included in the scoping review by two independent reviewers (MR and AF) using a standardized data extraction tool developed specifically for this review. Extracted data will encompass key information related to the participants, concept, context, study design, and main findings relevant to the review questions. The tool will undergo pilot testing by both reviewers to ensure consistency and reliability.

A draft version of the data extraction form is provided in Table 2. The tool may be refined and adapted as needed throughout the data extraction process. All modifications will be documented and reported in the final review. Any discrepancies between reviewers will be resolved through discussion or, if necessary, with the involvement of additional reviewers (JM and DV). When required, study authors will be contacted to obtain missing or supplementary information.

**Table 2:** Data extraction instrument, for primary studies.

Authors	
Year of publication	
Study design	
Aims/purpose	
Context	Country
	Setting
Participants	Group and size
	Age
	Gender
	Transplant indication
Concept	Time to mobilization after LT
	Type of mobilization
	Comparator (if applicable)
	Numbers of intervention
	Duration
	Frequency
	Outcomes
	Adverse event
Keys findings	

### Data analysis and presentation

The extracted data will be presented in various formats to address the research question, including tables and graphs.

These results will provide insights into the timing of initiation, mobilization techniques used, outcomes achieved, side effects observed, and barriers to early mobilization. Finally, a narrative summary will highlight gaps in the literature and outline future research directions.

### Protocol validation

The proposed scoping review will be conducted in accordance with the JBI methodology for scoping reviews [27]. It will be reported according to the Preferred Reporting Items for Systematic Reviews and Meta-Analyses extension for Scoping Reviews (PRISMA-ScR) checklist [26]. This protocol has been registered in Open Science Framework. No language or date restrictions will be applied. Study selection and data extraction will be performed independently by two reviewers.

### Limitations

While this protocol aims to provide a comprehensive overview of early mobilization following lung transplantation, several potential limitations must be acknowledged.

First, the choice of 7 days as a cut-off to define early mobilization may be open to discussion. In the literature, various thresholds have been proposed to distinguish early from late rehabilitation—ranging from 48 h to 72 h or even 7 days (references #16–18). We chose the 7-day mark because the complexity of care following LTx often delays mobilization, making it unfeasible in the immediate postoperative period. This criterion allows for the inclusion of studies in which mobilization begins a few days after surgery, without excluding relevant data.

Second, as there is currently no standardized protocol for initiating rehabilitation in the intensive care unit, we anticipate that the included studies will be of varying methodological quality and may lack control groups. This expected heterogeneity is a key reason for conducting a scoping review rather than a meta-analysis. Furthermore, the mobilization techniques are expected to be diverse, necessitating a rigorous and structured approach to map and interpret the available evidence.

It is also important to note that further limitations—particularly those concerning result interpretation and the variability of reported outcomes—will be addressed in the final article, once data extraction and synthesis are completed. This will allow for a more thorough discussion of the strengths and weaknesses of the existing evidence.

## CRediT author statement

**Matthieu Reffienna:** Conceptualization, Methodology, Writing - Original Draft. **Adéla Foudhaïli:** Methodology, Writing - Review & Editing. **Colin Sidre:** Methodology, Investigation, Writing - Review & Editing. **Damien Vitiello:** Conceptualization, Supervision, Writing - Review & Editing. **Jonathan Messika:** Conceptualization, Supervision, Writing - Review & Editing.

During the preparation of this work, the authors used ChatGPT (OpenAI, San Francisco, CA, USA) to improve the language and enhance readability. Following its use, the authors carefully reviewed and edited the content as necessary and take full responsibility for the final version of the publication.

## Declaration of competing interest

The authors declare the following financial interests/personal relationships which may be considered as potential competing interests: JM received congress reimbursement fees from Biotest/Grifols and Therakos, and received speaking fees honoraria from Biotest/Grifols and Therakos. No other financial disclosure was reported.

## Data Availability

No data was used for the research described in the article.

## References

[1] Roux A., Sage E., Cerf C., Le Guen M., Picard C., Hamid A.M. (2019 Feb). Évolution et progrès en transplantation pulmonaire : étude de la cohorte de 600 premiers patients transplantés pulmonaires à l’hôpital Foch. Rev Mal Respir.

[2] Khush K.K., Cherikh W.S., Chambers D.C., Harhay M.O., Hayes D., Hsich E. (2019 Oct). The international thoracic organ transplant registry of the international society for heart and lung transplantation: thirty-sixth adult heart transplantation report — 2019; focus theme: donor and recipient size match. J. Heart Lung TransPlant.

[3] Burgel P.R., Durieu I., Chiron R., Ramel S., Danner-Boucher I., Prevotat A. (2021 Jul). Rapid improvement after starting elexacaftor–tezacaftor–ivacaftor in patients with cystic fibrosis and advanced pulmonary disease. Am. J. Respir. Crit. Care Med..

[4] Totti V., Campione T., Mosconi G., Tamè M., Tonioli M., Gregorini M. (2020 Jun). Observational retrospective study on patient lifestyle in the pretransplantation and post-transplantation period in the emilia-romagna region. Transplant. Proc..

[5] Rozenberg D., Wickerson L., Singer L.G., Mathur S. (2014 Dec). Sarcopenia in lung transplantation: a systematic review. J Heart Lung Transplant.

[6] Langer D. (2015). Rehabilitation in patients before and after lung transplantation. Respiration.

[7] Quint E.E., Ferreira M., Van Munster B.C., Nieuwenhuijs-Moeke G., Te Velde-Keyzer C., Bakker S.J.L. (2023 Mar 25). Prehabilitation in adult solid organ transplant candidates. Curr. Transpl. Rep..

[8] Langer D., Burtin C., Schepers L., Ivanova A., Verleden G., Decramer M. (2012 Jun). Exercise training after lung transplantation improves participation in daily activity: a randomized controlled trial. Am J Transplant..

[9] Hatt K., Kinback N.C., Shah A., Cruz E., Altschuler E.L. (2017 Mar). A review of lung transplantation and its implications for the acute inpatient rehabilitation team. PM&R.

[10] Walsh J.R., Chambers D.C., Davis R.J., Morris N.R., Seale H.E., Yerkovich S.T. (2013). Impaired exercise capacity after lung transplantation is related to delayed recovery of muscle strength. Clin Transplant.

[11] Polastri M., Pehlivan E., Reed R.M., Eden A. (2024 Oct 31). Postoperative conditions of rehabilitative interest in lung transplantation: a systematic review. JYMS.

[12] Abrams D., Brodie D., Arcasoy S.M. (2017 Dec). Extracorporeal life support in lung transplantation. Clin. Chest Med..

[13] Vanhorebeek I., Latronico N., Van Den Berghe G. (2020 Apr). ICU-acquired weakness. Intensive Care Med..

[14] Fazzini B., Märkl T., Costas C., Blobner M., Schaller S.J., Prowle J. (2023 Jan 3). The rate and assessment of muscle wasting during critical illness: a systematic review and meta-analysis. Crit. Care.

[15] Van Aerde N., Meersseman P., Debaveye Y., Wilmer A., Gunst J., Casaer M.P. (2020 Jun). Five-year impact of ICU-acquired neuromuscular complications: a prospective, observational study. Intensive Care Med..

[16] Iwashyna T.J. (2012 Aug 15). Trajectories of recovery and dysfunction after acute illness, with implications for clinical trial design. Am. J. Respir. Crit. Care Med..

[17] Roeseler J., Sottiaux T., Lemiale V., Lesny M., Beduneau G., Bialais E. (2013 Mar). Prise en charge de la mobilisation précoce en réanimation, chez l’adulte et l’enfant (électrostimulation incluse). Réanimation.

[18] Schaller S.J., Scheffenbichler F.T., Bein T., Blobner M., Grunow J.J., Hamsen U. (2024 Jul 29). Guideline on positioning and early mobilisation in the critically ill by an expert panel. Intensive Care Med. [Internet].

[19] Paton M., Chan S., Serpa Neto A., Tipping C.J., Stratton A., Lane R. (2024 May). Association of active mobilisation variables with adverse events and mortality in patients requiring mechanical ventilation in the intensive care unit: a systematic review and meta-analysis. Lancet Respir. Med..

[20] Tarrant B.J., Holland A., Le Maitre C., Robinson R., Corbett M., Bondarenko J. (2018 Jul). The timing and extent of acute physiotherapy involvement following lung transplantation: an observational study. Physiotherapy Res. Intl..

[21] Song J.H., Park J.E., Lee S.C., Kim S., Lee D.H., Kim E.K. (2018 Aug 31). Feasibility of immediate in-intensive care unit pulmonary rehabilitation after lung transplantation: a single center experience. Acute Crit. Care.

[22] Mao L., Luo L., Wang D., Yu Y., Dong S., Zhang P. (2021 Mar). Early rehabilitation after lung transplantation with extracorporeal membrane oxygenation (ECMO) of COVID-19 patient: a case report. Ann. Transl. Med..

[23] Huebner L., Warmbein A., Scharf C., Schroeder I., Manz K., Rathgeber I. (2024 Apr 6). Effects of robotic-assisted early mobilization versus conventional mobilization in intensive care unit patients: prospective interventional cohort study with retrospective control group analysis. Crit. Care.

[24] Rossi V., Tammaro S., Santambrogio M., Retucci M., Gallo F., Crotti S. (2021 Nov 24). Physiotherapy approach after lung transplantation in a critically ill COVID-19 patient: a case report. Monaldi Arch. Chest Dis..

[25] Polastri M., Venturini E., Pastore S., Dell’Amore A. (2015 Sep). Do chest expansion exercises aid re-shaping the diaphragm within the first 72 hours following lung transplantation in a usual interstitial pneumonia patient?. PhysiOther Res. Int..

[26] Tricco A.C., Lillie E., Zarin W., O’Brien K.K., Colquhoun H., Levac D. (2018 Oct 2). PRISMA extension for scoping reviews (PRISMA-ScR): checklist and explanation. Ann. Intern. Med..

[27] Aromataris E., Lockwood C., Porritt K., Pilla B., Jordan Z. (2024). JBI manual for evidence synthesis. JBI [Internet].

